# Identification of Novel Serum Metabolic Biomarkers as Indicators in the Progression of Intravenous Leiomyomatosis: A High Performance Liquid Chromatography-Tandem Mass Spectrometry-Based Study

**DOI:** 10.3389/fcell.2021.695540

**Published:** 2021-07-08

**Authors:** Zhitong Ge, Penghui Feng, Zijuan Zhang, Jianchu Li, Qi Yu

**Affiliations:** ^1^Department of Ultrasound, State Key Laboratory of Complex Severe and Rare Diseases, Peking Union Medical College Hospital, Chinese Academy of Medical Science and Peking Union Medical College, Beijing, China; ^2^Department of Obstetrics and Gynecology, Peking Union Medical College Hospital, Chinese Academy of Medical Sciences, Peking Union Medical College, Beijing, China; ^3^Department of Pathology, Molecular Pathology Research Center, Peking Union Medical College Hospital, Chinese Academy of Medical Sciences, Peking Union Medical College, Beijing, China

**Keywords:** metabolomics, biomarkers, intravenous leiomyomatosis, progression, HPLC-MS/MS

## Abstract

**Background:**

Intravenous leiomyomatosis (IVL) is a rare estrogen-dependent neoplasm. However, identifiable and reliable biomarkers are still not available for clinical application, especially for the diagnosis and prognosis of the disease.

**Methods:**

In the present study, 30 patients with IVL and 30 healthy controls were recruited. Serum samples were isolated from these participants for further high performance liquid chromatography-tandem mass spectrometry (HPLC-MS/MS) analysis to study metabolomics alterations and identify differentially expressed metabolites based on orthogonal partial least-squares discriminant analysis (OPLS-DA). Subsequently, lasso regression analysis and a generalized linear regression model were applied to screen out hub metabolites associated with the progression of IVL.

**Results:**

First, 16 metabolites in the positive ion mode were determined from the 240 identifiable metabolites at the superclass level, with ten metabolites upregulated in the IVL group and the remaining six metabolites downregulated. Our data further proved that four metabolites [hypoxanthine, acetylcarnitine, glycerophosphocholine, and hydrocortisone (cortisol)] were closely related to the oncogenesis of IVL. Hypoxanthine and glycerophosphocholine might function as protective factors in the development of IVL (OR = 0.19 or 0.02, respectively). Nevertheless, acetylcarnitine and hydrocortisone (cortisol), especially the former, might serve as risk indicators for the disease to promote the development or recurrence of IVL (OR = 18.16 or 2.10, respectively). The predictive accuracy of these hub metabolites was further validated by the multi-class receiver operator characteristic curve analysis (ROC) with the Scikit-learn algorithms.

**Conclusion:**

Four hub metabolites were finally determined via comprehensive bioinformatics analysis, and these substances could potentially serve as novel biomarkers in predicting the prognosis or progression of IVL.

## Introduction

Intravenous leiomyomatosis (IVL) is a rare estrogen-dependent neoplastic disease (Low et al., 2012; Price et al., 2017), that was first described in 1896 (Marshall and Morris, 1959; Zhang Y. et al., 2016). In 1907, Durck documented and reported the first case of IVL involving the heart (Gui et al., 2016). It is characterized by invasive growth even though IVL appears to be benign histologically. The tumor originates from the venous wall of the uterus or the pelvic cavity outside the uterus, protrudes into the venous passage of the uterus or pelvis, invades through the iliac vein or ovarian vein, and extends to the inferior vena cava (Spanuchart et al., 2012). It can cause severe circulatory disturbance and syncope or sudden death once the tumor enters the right atrium or the right ventricle as well as the pulmonary artery through the tricuspid valve (Shi and Shkrum, 2018). If part of the tumor in the heart dislodges, this can lead to pulmonary embolism or cerebral infarction, which is life-threatening (Zhang et al., 2012; Knight et al., 2017; Kong et al., 2020). According to a single-center study of 30,757 Chinese patients who received treatment for hysteromyoma, the incidence of IVL disease among patients with uterine leiomyoma was 0.25% (Ma et al., 2016). Nevertheless, the rate of misdiagnosis and missed diagnosis of IVL before the operation is relatively high as the clinical symptoms and imaging findings are not specific. When IVL lesions are confined to the pelvic cavity without vascular invasion in the early stage, they may behave similarly to uterine leiomyoma (Yu et al., 2021). If the tumor invades into the inferior vena cava or heart in the later stage, it tends to be misdiagnosed as a primary cardiac tumor or venous thrombosis (Ribeiro et al., 2013; Kuklik et al., 2020). In clinical practice, surgical resection of the primary tumor, vena cava and right cardiac system tumor is the best choice (Deng et al., 2020). Studies have shown that the risk of postoperative recurrence of IVL is as high as 30% (Virzi et al., 2007), and the risk of postoperative recurrence in patients with large vein involvement is significantly higher than that in patients without it (Yu et al., 2016).

At present, an increasing number of studies have begun to focus on finding potential regulatory factors and biomarkers that affect the occurrence and progression of the disease, which are of great significance for the diagnosis, treatment and prognosis of IVL. Based on RNA sequencing analysis and reverse transcription-quantitative PCR, Wang W. et al. (2020) verified that IVL tended to be a solid tumor differing from uterine leiomyoma and found that *CDKN2A*, *BCL2A1*, and angiogenesis-related gene *CXCL8* might be new specific biomarkers of IVL. However, these findings remained to be further confirmed by larger samples (Wang W. et al., 2020). In another study, molecular analysis of 17 cases of IVL revealed that *MED12* mutations, *MSI* and *LOH* were inconsistent among patients with either uterine or extrauterine IVL. These findings suggested that IVL disease had a different molecular pathogenesis pattern from typical uterine leiomyoma (Lu et al., 2020). Other studies have detected 22q repeat deletions (66%) and complex copy number variants using array comparative genomic hybridization (Buza et al., 2014). Zhang et al. (2019) found that *HOXA13* was significantly downregulated in IVL when compared with myometrial tissue via RNA sequencing. Besides, the *Rb* pathway was proved to be involved in the pathogenesis of IVL disease (Ordulu et al., 2020). The above studies have initiated meaningful exploration of the mechanism of the occurrence and development of IVL, which are of great importance for deepening our understanding of the disease. However, most studies only explored the potential regulatory genes that affected the pathogenesis and prognosis of IVL and did not pay attention to the changes in a series of metabolites in patients. Thus, there have been no specific biochemical indicators for the diagnosis and prognosis of IVL.

Therefore, in the present study, we analyzed the difference in serum metabolomics between IVL patients and healthy controls based on high performance liquid chromatography-tandem mass spectrometry (HPLC-MS/MS). The core metabolic indicators affecting disease progression and recurrence were screened and identified, which provided a valuable theoretical basis for the development of new non-invasive methods for the diagnosis and prognostic intervention of IVL based on these biomarkers.

## Materials and Methods

### Experimental Design and Sample Collection

This study adhered to the Helsinki Declaration and the guidelines of the Clinical Practice Coordination Conference and was approved by the Ethics Committee of Peking Union Medical College Hospital (Ethics: JS-2654). All participants in the study signed informed consent forms. The disease group in this study included patients with IVL who underwent surgery at Peking Union Medical College Hospital from December 2011 to May 2020 and were followed up regularly. The study population was divided into groups: 30 healthy controls as a Co group, with half of them without uterine myoma (Co-no) and the rest with uterine myoma (Co-um); 30 patients with IVL were further classified into a non-recurrence group (IVL-no) or recurrence group (IVL-re) according to postoperative recurrence. There were 15 individuals in each of the above four subgroups.

In the Co-no group, the myometrium echo of these healthy women was uniform, there were no uterine myomas or pelvic space-occupying lesions, and the abdominal pelvic vein was confirmed by ultrasound examination. The Co-um group was characterized by a hypoechoic mass found in the mesometrium by ultrasound (maximum diameter ≥2 cm), the ultrasound pattern of which showed a typical vortex-like structure, and the abdominal and pelvic veins were confirmed by ultrasound. In the IVL-re group, a space-occupying lesion (≥1 cm) was found in the pelvic or abdominal veins (inferior vena cava and iliac veins) or the residual lesion was larger than the previous size on by at least two consecutive ultrasound examinations. If there were no space-occupying lesions in the pelvic cavity or blood vessels, patients were categorized into IVL-no group.

The admission criteria of the disease group were as follows: (1) abdominal and pelvic veins (parauterine reproductive vein, iliac vein, and inferior vena cava) or right atrium-occupying lesions observed in preoperative imaging or during surgery; (2) patients who received surgical treatment at Peking Union Medical College Hospital; (3) postoperative pathological diagnosis of IVL with vascular invasion; and (4) age ≥18 years. The exclusion criteria of the disease group were as follows: (1) incomplete clinical data; (2) pregnant and lactating women; (3) people with intellectual disability or an inability to take care of themselves; (4) patients who were not willing to participate in this study; and (5) patients with other malignant tumors. At the same time, we recruited women to the healthy control group. The criteria for the healthy control group were as follows: (1) age ≥18 years old; (2) no history of other malignant tumors; (3) non-pregnant and lactating women; and (4) no previous history of hysteromyomectomy or hysterectomy-related gynecological surgery.

First, ultrasound examinations were performed on all subjects by senior doctors, results were read independently, and inconsistent results were resolved after discussion. All participants’ abdominal and pelvic blood vessels were examined on an empty stomach, and they were advised to retain their urine properly so that their uterine and double appendices could be properly evaluated. For the patients in the IVL group, the first step was to check the patency of the large abdominal vessels, including the inferior vena cava and bilateral iliac veins, followed by observing whether there was a space-occupying lesion within the pelvic cavity. In the Co group, inferior vena cava, iliac vein and gynecological ultrasonography were performed to confirm that there was no space-occupying lesion in the abdominal and pelvic blood vessels and whether there was myoma in the uterus. The location and size of the largest leiomyoma were recorded simultaneously if uterine myoma was present. After each patient finished the ultrasound examination, the venous blood was collected from the cubital vein for further metabolomics tests.

### Sample Preparation and LC-MS/MS Analysis

Serum was isolated from fasting blood samples after centrifugation for 15 min at 2,000 × *g*. Each aliquot (150 μL) of the serum samples was maintained at −80°C until UPLC-Q-TOF/MS analysis. After thawing, the serum aliquots were mixed with 400 μL of precooled methanol/acetonitrile (1:1, v/v) to remove the protein, followed by centrifugation for 15 min at 14,000 × *g*. The supernatant was further dried through vacuum centrifugation and redissolved in 100 μL acetonitrile/water liquor (1:1, v/v) for LC-MS analysis, which was carried out with an UHPLC (1290 Infinity LC, Agilent Technologies) coupled to a quadrupole time-of-flight (AB SCIEX TripleTOF 6600). For HILIC separation, samples were detected using an ACQUITY UPLC BEH column (Waters, Ireland). In ESI either positive or negative modes, the mobile phase included solution A (25 mM ammonium acetate and 25 mM ammonium hydroxide in water) and solution B (acetonitrile). The gradient condition was as follows: 85% solution B for 1 min, linearly reduced to 65% in 11 min, and then reduced to 40% in 0.1 min and sustained for 4 min, followed by elevation to 85% in 0.1 min, with a 5 min reequilibration period employed. The ESI source conditions were executed as follows: ion source gas1 (Gas1) of 60, ion source gas2 (Gas2) of 60, curtain gas (CUR) of 30, source temperature of 600°C, and ion spray voltage floating (ISVF) ± 5,500 V. The product ion scan was finally obtained based on information-dependent acquisition (IDA) with the high sensitivity mode selected.

The raw MS data (wiff.scan files) were transformed into MzXML files with ProteoWizard MSConvert before being imported into XCMS software. Collection of algorithms of the metabolite profile annotation (CAMERA) was applied to annotate the isotopes and adducts. For the extracted ion features, only the variables with no less than 50% of the non-zero measurement values in at least one group were screened out. Compound identification of metabolites was carried out in comparisons of accuracy m/z value (<25 ppm), and MS/MS spectra.

### Statistical Analysis

For baseline information, data were expressed as numbers (percentages) for categorical variables or medians (upper quartile and lower quartile) for continuous variables based on the normality test of data distribution. The Kruskal–Wallis non-parametric test was performed for comparisons among different specialties. For LC-MS/MS data, after normalization to the total peak intensity, the processed data were analyzed by R package (ropls), where it was subjected to orthogonal partial least-squares discriminant analysis (OPLS-DA). The cross-validation and response permutation testing were used to evaluate the robustness of the model. The variable importance in the projection (VIP) value of each variable in the OPLS-DA model was calculated to represent its contribution to the classification. Metabolites with a VIP value >1 were further subjected to Student’s *t*-test or Mann-Whitney *U* test at the univariate level to measure the significance of each metabolite, and *P*-values less than 0.05 were considered statistically significant.

## Results

### Baseline Characteristics of the Subjects

This study involved 30 IVL patients and 30 healthy controls, whose median age was 49.0 and 49.5 years, respectively (*P* = 0.25). Additionally, the age at menarche did not differ between groups. The symptoms of the IVL patients were atypical, mainly including lower limb edema (*n* = 4), flustered shortness of breath (*n* = 8), and lumbago or back pain (*n* = 4). Some of the patients were asymptomatic, and only a few people complained about abdominal masses, hypermenorrhagia, a ventral belly, or even syncope. In most cases, the average diameter of the pelvic mass varied by approximately 8.3 cm. All IVL patients in this study had a history of uterine fibroids. Likewise, more than two-thirds of these patients have undergone uterine surgeries. It is worth noting that the extension route of IVL was concentrated in the left or right iliac veins and right gonadal vein. Furthermore, the extensions of involvement chiefly consisted of the right ventricle (*n* = 4), right atrium (*n* = 14), and infrarenal inferior vena cava (IVC) (*n* = 4), with shapes categorized as either cast or luminal. Regarding the IVL staging operation, 23 patients underwent a surgery with stage I disease, and the remaining patients underwent a surgery with stage II disease. In addition, most of these patients received complete resection except for four patients who had an intravascular residue and 1 patient who had a pelvic residue. More importantly, half of the patients experienced recurrence after surgery, with the lesions primarily presenting within the blood vessel, pelvis, or both locations. The detailed information was presented in Table 1.

**TABLE 1:** Baseline characteristics of the involved patients.

Characteristic	Groups		*P*-value
	IVL patients (*n* = 30)	Normal controls (*n* = 30)	
**Age (years)**	49.0 (45.0∼53.5)	49.5 (43.0∼52.0)	0.25
**Age of menarche (years)**	14.0 (13.0∼15.0)	15.0 (13.0∼16.0)	0.07
**Symptoms**			–
Lower limb edma	4 (13.3)	–	
Flustered shortness of breath	8 (26.7)	–	
Abdominal mass	1 (3.3)	–	
Lumbago and back pain	4 (13.3)	–	
Hypermenorrhagia	2 (6.7)	–	
Ventral belly	2 (6.7)	–	
Syncope	2 (6.7)	–	
Asymptomatic	7 (23.3)	–	
**Pelvic mass (cm)**	8.3 (0.0∼12.2)	–	–
**History of uterine fibroids**	30 (100)	–	–
**Uterine surgery history**	22 (73.3)	–	–
**Route of extension**			–
Left iliac vein	4 (13.3)	–	
Right iliac vein	24 (80.0)	–	
Left gonadal vein	1 (3.3)	–	
Right gonadal vein	4 (13.3)	–	
Left parauterine vein	1 (3.3)	–	
Right parauterine vein	1 (3.3)	–	
**Extent of involvement**			–
Right ventricle	4 (13.3)	–	
Right atrium	14 (46.7)	–	
Retrohepatic IVC	3 (10.0)	–	
Infrarenal IVC	4 (13.3)	–	
Distal IVC	1 (3.3)	–	
Left iliac vein	1 (3.3)	–	
Right iliac vein	3 (10.0)	–	
**Shape of IVL**			–
Cast	27 (90.0)	–	
Luminal	3 (10.0)	–	
Thread-like	0 (10.0)	–	
Mixed	0 (10.0)	–	
**Staging surgery**			–
I	23 (76.7)	–	
II	7 (23.3)	–	
**Postoperative residual lesions**			–
No	25 (83.3)	–	
Intravascular residue	4 (13.3)	–	
Pelvic residue	1 (3.3)	–	
**Site of recurrence**			
Intravascular	4 (13.3)		
Pelvic	9 (30.0)		
Both	2 (6.7)		

*IVL, intravenous leiomyomatosis; IVC, inferior vena cava. Data were expressed as number (percentage) for categorical variables or median (upper quartile and lower quartile) for continuous ones. Kruskal–Wallis non-parametric test was performed for comparisons among different specialties.*

### Differential Metabolites Between IVL Patients and Healthy Controls

**FIGURE 1:**
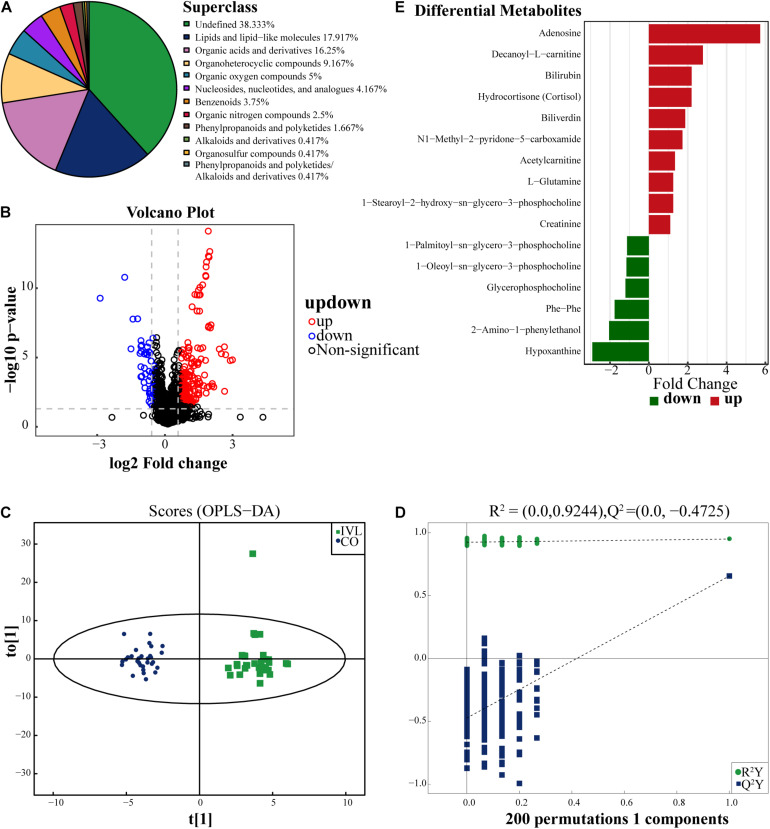
Identification of differentially expressed metabolites. **(A)** The pie chart displayed all the metabolites identified in this project in either positive or negative ion modes, and their chemical attribution. **(B)** Volcano diagram revealed the changes between the IVL group and the healthy subjects. The red dots on the right side of the figure represented the upregulated metabolites, the blue dots on the left side meant the downregulated metabolite, and the black dots referred to non-significant difference. *X*-axis corresponded to log2 fold change and *y*-axis corresponded to –log10 *P*-value. **(C)** Score plot of OPLS-DA for IVL and control groups, indicating the separation degree between the two groups. **(D)** A permutation test conducted with 200 randomly initiated permutations in an OPLS-DA model by cross-validation. T[1] referred to principal component 1, to[1] represented principal component 2, and the ellipse was 95% confidence interval. The dots in the same color meant each biological repetition within the group, and the distribution state of the dots reflected the degree of difference between groups and within groups. **(E)** The bar chart displayed 16 significant metabolites between IVL patients and control group (VIP > 1, *P* < 0.05). The *x*-axis represented the fold change of differential expression of these metabolites.

As exhibited in Figure 1A, a total of 240 kinds of metabolites at the superclass level (above level two, including undefined metabolites) were finally identified in this project and detected in the positive and negative ion modes. These metabolites were classified according to their chemical compound attributes. Our results demonstrated that most of these identifiable metabolites were lipids or lipid-like molecules (17.92%), organic acids or derivatives (16.25%) and organoheterocyclic compounds (9.167%). Besides, some organic oxygen compounds, nucleosides, nucleotides, or analogs, benzenoids, organic nitrogen compounds, and phenylpropanoids or polyketides were detected in the serum specimens as well. To analyze the differences between the IVL and healthy control groups in the positive ion mode, we used univariate statistical analysis methods, including fold change (FC) analysis and Student’s *t*-test or the Mann-Whitney *U* test. Among these metabolites, the differentially expressed metabolites (DEMs) were filtered out and displayed as the volcano plot in Figure 1B, with FC > 1.5 and *P*-value < 0.05. In the volcano plot, the upregulated and downregulated metabolites were marked in red or blue, respectively. Our data indicated that these DEMs were classified into lipids or lipid-like molecules, nucleosides, nucleotides or analogs, and organoheterocyclic compounds.

In order to screen out the metabolites correlated with the oncogenesis of IVL, OPLS-DA, a supervised discriminant analysis method, was applied to distinguish the two groups of samples by establishing the discriminant model according to the metabolites. As shown in Figure 1C, the two groups were well separated from each other in positive ion mode based on the scores. So as to avoid overfitting of the supervised model in the modeling process, the permutation test was adopted to ensure the efficacy of the model. Figure 1D demonstrated that the *R*^2^ and *Q*^2^ values of the random model gradually decreased as the permutation retention lessened, which implied that the robustness of the model was acceptable and that the model established in this study had a favorable degree of fitting and predictability with R^2^Y = 0.95 and *Q*^2^ = 0.66 of the original model. Based on the analysis, 16 metabolites in the positive ion mode were ultimately identified when meeting the screening criteria in this experiment: VIP > 1 and *P*-value < 0.05, as displayed in Figure 1E. Among these metabolites, 10 of them were upregulated in the IVL group, including adenosine, decanoyl-L-carnitine, bilirubin, hydrocortisone (cortisol), and biliverdin, etc. Conversely, 1-palmitoyl-sn-glycero-3-phosphocholine, 1-oleoyl-sn-glycero-3-phosphocholine, glycerophosphocholine, phe-phe, 2-amino-1-phenylethanol, and hypoxanthine were downregulated when compared with the Co group.

Comparisons of the total ion chromatograms (TICs) of quality control (QC) samples in both the positive and negative ion modes were performed. Our results manifested that the response intensity and retention time of each chromatographic peak basically overlapped, indicating that the variation caused by the error of the instrument was negligible throughout the whole experiment (Supplementary Figures 1A,B). Additionally, the comprehensive evaluation results of the total sample by principal component analysis (PCA) were expressed in Supplementary Figures 1C,D, and signified that, in this experiment, the stability and repeatability of the investigation, and the reliability of the data quality were sufficient for further analysis.

### Hierarchical Cluster Analysis and KEGG Pathway Enrichment

**FIGURE 2:**
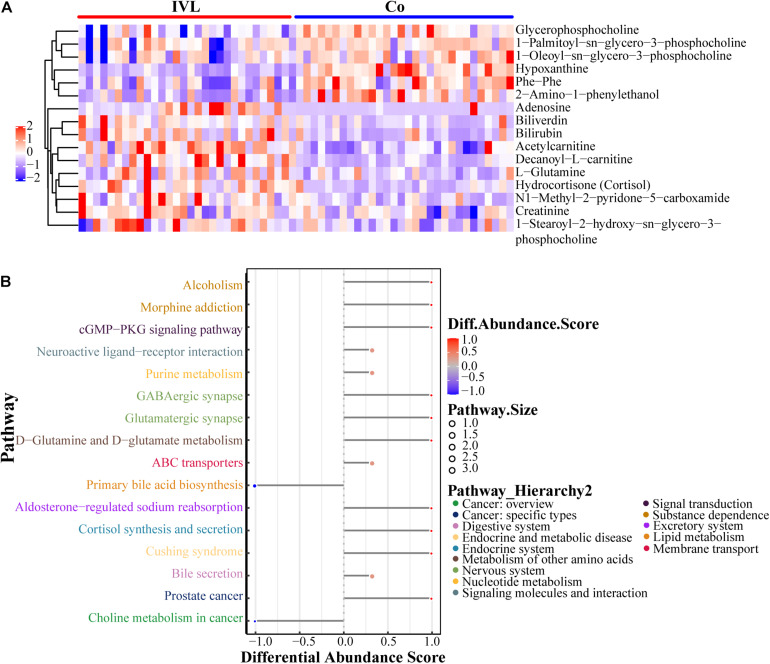
Characteristics of 16 identified metabolites. **(A)** Heatmap visualization of metabolomics data with hierarchical clustering analysis in the positive ion mode. Colors reflected the relative content of metabolites in serum. The more similar the colors were, the more similar the expression pattern. The panel on the right indicated the different metabolites. Each blockage meant a sample of either the IVL or Co groups. **(B)** The differential abundance (DA) score of differential metabolic pathways. *Y*-axis represented the name of the differential pathways, and *X*-axis coordinates meant the DA score. DA score was defined as the total change of all metabolites in the metabolic pathway. A score of 1 indicated an upregulated expression trend for all identified metabolites in the pathway, while –1 was exactly the opposite. The length of the line segment represented the absolute value of DA score; the size of the dot at the end of the line segment referred to the number of metabolites in the pathway; the larger the dot was, the greater the number of metabolites. The color depth of the line segment and dot was proportional to DA score.

To fully and intuitively reveal the relationships and differences between different samples, hierarchical clustering analysis (HCA) was conducted. Metabolites clustered in the same cluster were characterized by similar expression patterns and might have similar functions or participate in the same metabolic processes or cellular pathways. The HCA results included the above 16 significant DEMs (VIP > 1, *P* < 0.05), as shown in Figure 2A. It was noticed that the two groups shared completely different metabolic patterns with six upregulated metabolites chiefly enriched in the Co group, including glycerophosphocholine, 1-palmitoyl-sn-glycero-3-phosphocholine, 1-oleoyl-sn-glycero-3-phosphocholine, hypoxanthine, phe-phe, and 2-amino-1-phenylethanol. In contrast, the remaining ten metabolites were abundant in the IVL group. Subsequently, in order to capture the average and overall variation of the above 16 DEMs in specific pathways, the analysis of the metabolic changes based on differential abundance (DA) score was introduced into this study. As shown in Figure 2B, these metabolites were mainly involved in the cGMP-PKG signaling pathway, neuroactive ligand-receptor interaction, GABAergic synapse, glutamatergic synapse, D-glutamine or D-glutamate metabolism, purine metabolism, and cortisol synthesis or secretion, as well as choline metabolism in cancer. All differential metabolic pathways were further classified and assigned according to their previous pathway hierarchy, and these metabolites were principally engaged in cancer, endocrine or metabolic disease, metabolism of other amino acids or nucleotides, signaling molecule interaction or transduction, and lipid metabolism, along with membrane transport.

### Screening of Metabolites Based on Lasso Regression Analysis

**FIGURE 3:**
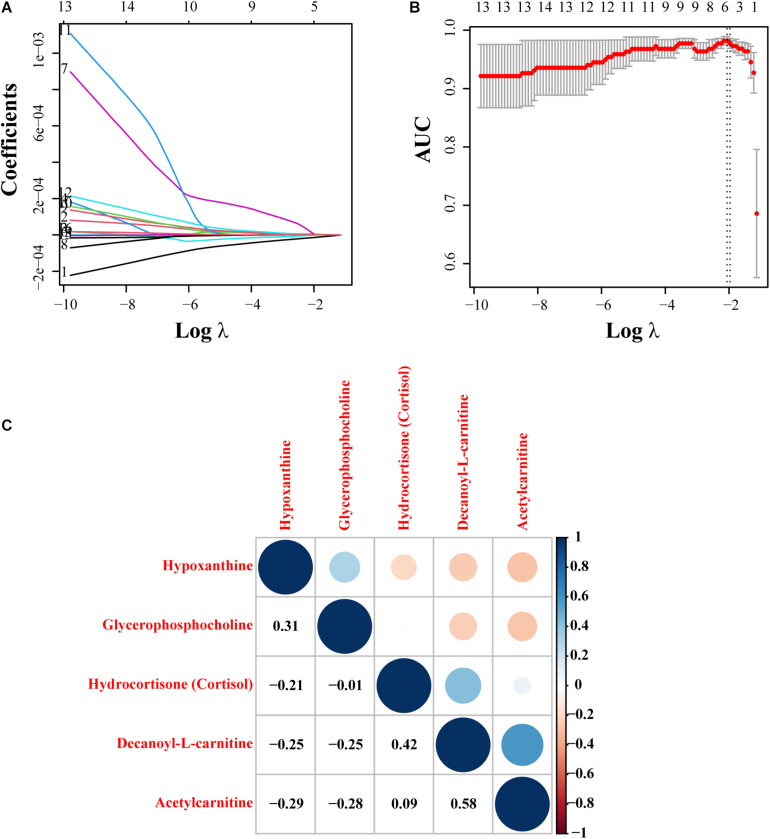
Lasso regression model construction and correlation analysis. **(A)** The process of fitting the lasso regression model. Each curve represented a metabolite. **(B)** Partial likelihood deviance was calculated by the cross-validation for the best λ to determine the minimum mean cross-validated error. Red dots and solid vertical lines referred to partial likelihood deviance and corresponding 95% CI, respectively. In addition, the left and right dotted vertical lines represented the λ value with minimum cvm (namely, lambda.min) and the largest value, whose error was within one standard error of the minimum (lambda.1se). **(C)** The correlation figure revealed the correlation of expression pattern of five selected metabolites. Red represented a positive correlation, and blue showed a negative correlation. The size of the point was proportional to the correlation coefficient.

**FIGURE 4:**
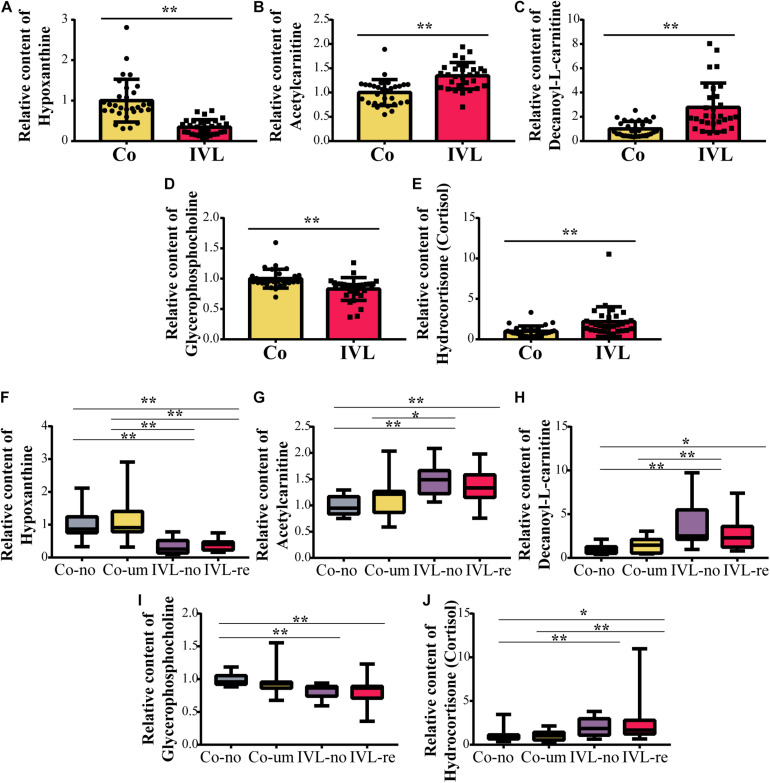
The relative content of metabolites among groups. **(A–E)** Boxplots of the differentially expressed metabolites between the IVL and control groups (**P < 0.01), including hypoxanthine, glycerophosphocholine, hydrocortisone (cortisol), decanoyl-L-carnitine, and acetylcarnitine. **(F–J)** Comparisons of the expression of five metabolites as mentioned above among the Co-no, Co-um, IVL-no, and IVL-re groups (*P < 0.05, **P < 0.01).

Among the metabolites screened above, lasso regression analysis was carried out and five metabolites were determined, which were further validated based on the cross-validation, including hypoxanthine, glycerophosphocholine, hydrocortisone (cortisol), decanoyl-L-carnitine, and acetylcarnitine (Figures 3A,B). To interpret the metabolic correlation among all DEMs, correlation analysis was performed, which was meaningful for further understanding the mutual regulation of metabolites. Metabolites with expression correlations might jointly participate in the biological processes, namely, functional correlations. On the basis of this analysis, we found that hypoxanthine exhibited a positive relationship with glycerophosphocholine (Cor = 0.31), and decanoyl-L-carnitine also shared a positive correlation with either hydrocortisone (cortisol) or acetylcarnitine (Cor = 0.42, 0.58, respectively). Instead, acetylcarnitine presented a negative trend with the abundance of both hypoxanthine and glycerophosphocholine (Figure 3C). According to the expression levels of these five metabolites between the IVL and Co groups, we discovered that hypoxanthine and glycerophosphocholine were significantly downregulated in the IVL group, while hydrocortisone (cortisol), decanoyl-L-carnitine, and acetylcarnitine were upregulated in the IVL group (Figures 4A–E). To investigate the roles of these metabolites in the progression of IVL, all samples were further classified into four groups as mentioned above in accordance with the presence of uterine leiomyoma and recurrence. It appeared that the relative content of hypoxanthine in the Co-no and Co-um groups significantly differed from that in the IVL-no and IVL-re groups (*P* < 0.01) (Figure 4F). Regarding acetylcarnitine and decanoyl-L-carnitine in Figures 4G,H, they were elevated in the IVL-no group when compared with the healthy controls. Besides, the levels of these metabolites were significantly different in the IVL-re group in comparison with the Co-no group. Glycerophosphocholine showed a tendency of declining consistency as the disease progressed even though we only observed a noticeable difference in the IVL-no and IVL-re groups when compared with the Co-no group but not when compared with the Co-um group (relative content = 1.00 ± 0.10, 0.95 ± 0.19, 0.82 ± 0.11, 0.80 ± 0.24 for each group, respectively) (Figure 4I). Conversely, a gradual upward trend was observed in hydrocortisone (cortisol) except that there seemed to be no statistical significance regarding the comparison between the IVL-no and Co-um groups, as suggested in Figure 4J (relative content = 1.00 ± 0.75, 1.08 ± 0.62, 1.99 ± 0.96, 2.58 ± 2.54 for each group, respectively).

### Determination of Hub Metabolites Associated With the Progression of IVL

**FIGURE 5:**
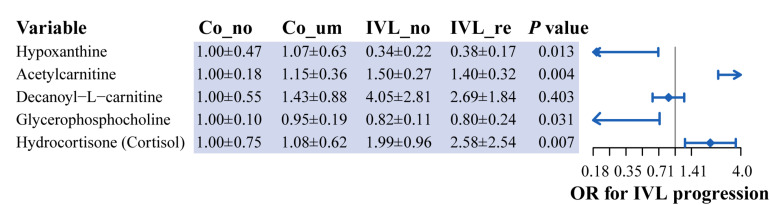
Identification of hub metabolites based on generalized linear regression model. Forest plot displayed the odds ratio of five metabolites in the progression of IVL. *P*-value was calculated by generalized linear regression model, adjusted for the relative content of these metabolites.

Given the mutual interaction of the metabolites, we further used a generalized linear regression model (GLM) to find the hub metabolites that were associated with the progression of IVL with the relative content of the five metabolites. Our data proved that four metabolites [hypoxanthine, acetylcarnitine, glycerophosphocholine, and hydrocortisone (cortisol)] were closely related to the progression of IVL, as displayed in Figure 5. Hypoxanthine and glycerophosphocholine might function as independent protective factors in the progression of the disease (OR = 0.19 or 0.02, respectively); nevertheless, acetylcarnitine and hydrocortisone (cortisol), especially the former, might act as hazardous indicators or risk factors for the disease to promote the progression of IVL (OR = 18.16 or 2.10, respectively). These results implied that these four metabolites were promising factors for future prediction of the prognosis and progression of IVL.

### Validation of the Prediction of the Hub Metabolites by ROC Analysis

**FIGURE 6:**
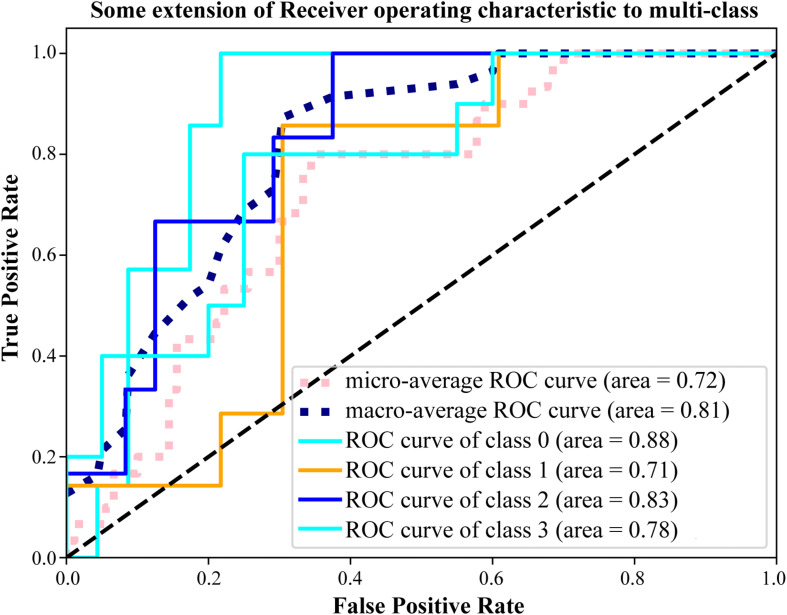
Validation the prediction accuracy of the model. ROC analysis of the four hub metabolites by micro and macro-average ROC machine-learning algorithms for multi-class variables. Class 0 ∼ 3 represented the Co-no, Co-um, IVL-no, and IVL-re groups, respectively. AUC value was applied to characterize the model performance.

To confirm the prediction accuracy of the four hub metabolites mentioned above, we further performed the receiver operator characteristic curve analysis using a Python module, Scikit-learn^1^. Two comprehensive state-of-the-art machine-learning algorithms were introduced in the present study, including micro-average ROC (calculating metrics globally by considering each element of the label indicator matrix as a label) and macro-average ROC (calculating metrics for each label, and finding their unweighted mean). Our results indicated that these four metabolites functioned well in distinguishing cases from distinct pathological statuses with the area under the curve (AUC) to be 0.72 or even 0.81 for the results of micro or macro-average ROC analyses, respectively (Figure 6). Additionally, it was worth noting that the four hub metabolites exhibited a reliable discrimination to recognize patients with uterine leiomyoma or intravenous leiomyomatosis from normal controls (AUC value = 0.88). These findings signified the predictive value of the model established by the four hub metabolites.

## Discussion

With the rapid development of modern biological analysis technology, metabolomics has been successfully applied in many fields, such as cancer research (Tayanloo-Beik et al., 2020). Metabolomics is one of the “omics” techniques, and is complementary to genomics, transcriptomics, and proteomics (Xuan et al., 2020). Metabolites are defined as small organic and low-molecular-weight compounds (<1,500 Dalton), which are the final products during the metabolic process (Alonso et al., 2015). The study of metabolites helps to identify metabolic pathways that are activated or dysfunctional in patients (Christodoulou et al., 2020). At the molecular level, metabolomics adopts novel biomarkers to explore the underlying mechanisms of disease development (Wang et al., 2011). A recent study discovered that histidinyl-lysine, docosahexaenoic acid and lysoPC combined with CA199, based on metabolomics analysis, showed high sensitivity and specificity in detecting patients with pancreatic ductal adenocarcinoma, indicating that these metabolites could be used as potential indicators to distinguish pancreatic ductal adenocarcinoma disease from normal controls (Zhang et al., 2020). Additionally, another team also proved that increased levels of circulating anti-Mullerian hormone (AMH) in premenopausal women were associated with elevated risks of breast cancer (Eliassen et al., 2016). A study involving 101 unstable angina pectoris patients screened out a series of DEMs, as new biomarkers in the identification of the illness (Yao et al., 2017). Similarly, Farshidfar et al. (2016) also tested some biomarkers that could achieve early detection or even preliminary staging information for colorectal cancer. The above studies demonstrated that metabolomics plays a vital role in the diagnosis and prognosis of diseases. However, there have been no relevant studies focusing on metabolic differences in patients with IVL.

In this study, 30 IVL patients and 30 healthy controls were recruited, and the program yielded a total of 240 significantly different metabolites at the superclass level, which were prevailingly characterized as lipids or lipid-like molecules, organic acids or derivatives, organoheterocyclic compounds, and organic oxygen compounds. Subsequently, we identified several metabolites associated with IVL based on OPLS-DA and univariate analysis. Further identification via GLM was performed, and four hub metabolic markers were finally screened out, namely, hypoxanthine, acetylcarnitine, glycerophosphocholine, and hydrocortisone (cortisol), which could distinguish IVL patients from healthy subjects. These metabolites were involved in purine metabolism, cortisol synthesis or secretion, and choline metabolite in cancer on the basis of enrichment analysis. Subsequently, the micro and macro-average ROC machine-learning algorithms were applied in this study to validate the prognostic reliability of the hub metabolite. What made sense was that the ROC analysis confirmed the discrimination of the model to recognize the corresponding disease conditions of these included cases. Particular, it performed well to distinguish patients with uterine leiomyoma or intravenous leiomyomatosis from healthy subjects.

Hypoxanthine is a purine, engaged in the metabolism of adenine and guanine, and therefore, in the synthesis of the corresponding nucleosides (Barberini et al., 2019). Hypoxanthine is known to be related to various cancers. A targeted study constructed by Yoo et al. (2010) revealed that the downstream metabolites, hypoxanthine and xanthine, were reduced in non-Hodgkin’s lymphoma, which were likely to be defined as suitable markers for non-Hodgkin’s lymphoma, with AUROC values of 0.85 and 0.83, respectively. Another study also elucidated the underlying mechanisms of the low content of hypoxanthine in urine samples of patients with hepatocellular carcinoma (Wu et al., 2009). Likewise, a research focusing on cerebrospinal fluid-based metabolomics, suggested that it was downregulated in patients with lung adenocarcinoma with brain metastasis compared with controls (Wang F.X. et al., 2020). In previous reports, the potential correlation between the reduced hypoxanthine levels and cancers such as colorectal cancer (Long et al., 2017), non-Hodgkin lymphoma (Yoo et al., 2010), and gastric cancer (Jung et al., 2014) has already been explained; in other words, increased synthesis of DNA (adenine utilization) in hyperproliferative tissues might be responsible for the decreased level of hypoxanthine. Consistent with previous studies, in the present research, the level of hypoxanthine was significantly lower in the IVL groups than in the control group (*P*-value = 0.013). As a substrate and nitrogen source, the alteration in hypoxanthine levels shown in our study was possibly associated with the higher invasive probability of the tumor cells.

Acetylcarnitine is an acetylated form of L-carnitine, mainly used for energy production by transporting activated long-chain fatty acids from the cytosol into mitochondria (Lee et al., 2020). Long-chain acetylcarnitine was reported to be an essential source of energy production in cancer cells (Linher-Melville et al., 2011). In recent years, acetylcarnitine has attracted much attention in tumor research (Fong et al., 2011). Several studies have carried out metabolomics analysis to compare patients with hepatocellular carcinoma and healthy individuals. The findings revealed that it was increased in serum of patients with hepatocellular cancer (Ladep et al., 2014; Li et al., 2017; Lee et al., 2020). Lu et al. (2016) also suggested that serum acetylcholine could be regarded as a new indicator of hepatocellular carcinoma. In addition, differences in acetylcarnitine levels were observed among different breast cancer subtypes (Fan et al., 2016). Conversely, given that advanced stages of hepatocellular carcinoma usually impelled patients to be prone to cachexia, serum acetylcarnitine levels declined accordingly for reduced carnitine synthesis in hepatocellular carcinoma patients (Lee et al., 2020), which was commonly seen in the terminal phase of patients with digestive system neoplasms (Malaguarnera et al., 2006). Acetylcarnitine in our study was higher in the IVL group than in the control group, and it functioned as a hazardous factor in the progression of IVL. Based on these theories, it was reasonable to believe that acetylcarnitine became elevated as the disease progressed by promoting energy production in the lesions.

Glycerophosphocholine is one of the intermediates of choline metabolism (Kinross et al., 2009). Abnormal choline phospholipid metabolism has been verified to be associated with carcinogenesis and tumor progression (Zhang X. et al., 2016). Besides, variations have been found in choline phospholipid metabolism, which could be observed among several types of cancers (Glunde et al., 2011). Similar to previous studies, our data showed that IVL patients had lower levels of glycerophosphocholine, a metabolite of choline, than the healthy controls, suggesting that abnormal choline and carbon metabolism might contribute to oncogenesis. As reported, when choline is oxidized to betaine (trimethylglycine), it participates in methylation, which is not only necessary for the methionine/homocysteine cycle but also plays a central role in choline-mediated carbon metabolism (it donates a methyl group for methionine and dimethylglycine remethylation of homocysteine) (Zhang X. et al., 2016). Choline can also be metabolized by intestinal bacteria to produce trimethylamine, which is then further transformed into trimethyl-amine N-oxide (Glunde et al., 2006). Compared with normal tissues, tumor tissues tended to present an abnormal choline phospholipid metabolic spectrum, characterized by abnormally high levels of choline-containing compounds. This eventually led to a decrease in these compounds in blood.

Regarding hydrocortisone (cortisol), it is the major endogenous glucocorticoid secreted by the adrenal cortex (Marik, 2009). As a glucocorticoid receptor agonist, hydrocortisone is involved in protein catabolism, gluconeogenesis, capillary wall stability, renal excretion of calcium, and suppression of immune or inflammatory responses. Furthermore, studies have validated that cortisol also exhibits special significance in some tumors; that is, as cancer progresses, cortisol secretion increases abnormally in cancers such as breast cancer, lung cancer and oral squamous cell carcinoma, which is not relative to the tumor tissue type (Lichter and Sirett, 1975; van der Pompe et al., 1996; McEwen et al., 1997; Lissoni et al., 2007; Sharma et al., 2018). In our experiment, cortisol levels could distinguish the IVL group from the control group, and the cortisol concentration in the IVL-re group was significantly higher than that in the IVL-no group, which revealed that cortisol might be associated with the recurrence of IVL.

Here, metabolomics and comprehensive bioinformatics analyses were combined to distinguish metabolites between patients with IVL and healthy controls. The aim was to determine the metabolic characteristics of IVL and improve the understanding of IVL development and the related prognosis. However, the limitations of the current study need to be pointed out either, that more cases of IVL patients should be recruited from multiple clinical centers to validate the reliability and feasibility of the model in the future application. Besides, it really makes much sense to distinguish the potential alteration or similarity in the metabolic hallmarks between solid tissues and serum samples from IVL patients given that tumors are characterized by peculiar internal environment and metabolic patterns.

## Conclusion

We found that metabolomics based on HPLC-MS/MS could efficaciously differentiate IVL patients from healthy subjects. This study characterized the serum metabolomics pattern of IVL. Ultimately, we developed a four-metabolite-based panel that was closely correlated with the progression of IVL. Among them, hypoxanthine, and glycerophosphocholine performed as underlying protective indicators, which decreased as the disease progressed. Conversely, acetylcarnitine and hydrocortisone (cortisol) were proved to be risk factors, augmenting with the oncogenesis and recurrence of IVL, further confirmed based on the multi-class ROC analysis. Thus, the abnormal metabolism and relevant metabolite differences in this study provided valuable evidence for developing novel non-invasive methods concerning the diagnosis and prognosis of IVL based on these potential biomarkers.

## Data Availability Statement

The datasets presented in this study can be found in online repositories. The names of the repository/repositories and accession number(s) can be found below: MetaboLights (https://www.ebi.ac.uk/metabolights/). The identifier of this study is MTBLS2714.

## Ethics Statement

The studies involving human participants were reviewed and approved by the Ethics Committee of Peking Union Medical College Hospital (Ethics: JS-2654). The patients/participants provided their written informed consent to participate in this study.

## Author Contributions

ZG and PF: software, data curation, formal analysis, and visualization. ZG, PF, and ZZ: writing-original draft preparation and writing-review and editing. JL and QY: conceptualization and design, and administration and funding acquisition. All authors contributed to the article and approved the submitted version.

## Conflict of Interest

The authors declare that the research was conducted in the absence of any commercial or financial relationships that could be construed as a potential conflict of interest.

## Footnotes

1 https://scikit-learn.org/

## Supplementary Material

Supplementary Figure 1 Quality control analysis of the project. **(A,B)** Total ion chromatogram of all samples along with quality controls in positive and negative modes, respectively. The abscissa represented the retention time of each chromatographic peak, and the ordinate represented the peak intensity. **(C,D)** Principal component analysis of all samples PCA in the positive and negative modes, respectively.

## Abbreviations

IVL: intravenous leiomyomatosis
HPLC-MS/MS: high performance liquid chromatography-tandem mass spectrometry
OPLS-DA: orthogonal partial least-squares discriminant analysis
Co-no: control group without uterine myoma
Co-um: control group with uterine myoma
IVL-no: IVL group without recurrence
IVL-re: IVL group with recurrence
ISVF: ion spray voltage floating
IDA: information dependent acquisition
CAMERA: collection of algorithms of metabolite profile annotation
VIP: variable importance in the projection
IVC: inferior vena cava
DEMs: differentially expressed metabolites
TIC: comparisons of total ion chromatogram
QC: quality control
PCA: principal component analysis
HCA: hierarchical clustering analysis
DA: differential abundance
GLM: generalized linear regression model
AMH: anti-Mullerian hormone.
