# Supplementary figures and images for: Identification of Novel Serum Metabolic Biomarkers as Indicators in the Progression of Intravenous Leiomyomatosis: A High Performance Liquid Chromatography-Tandem Mass Spectrometry-Based Study
Source: Front Cell Dev Biol. 2021 Jul 8;9:695540. doi: 10.3389/fcell.2021.695540 (PMC8297591; doi:10.3389/fcell.2021.695540)

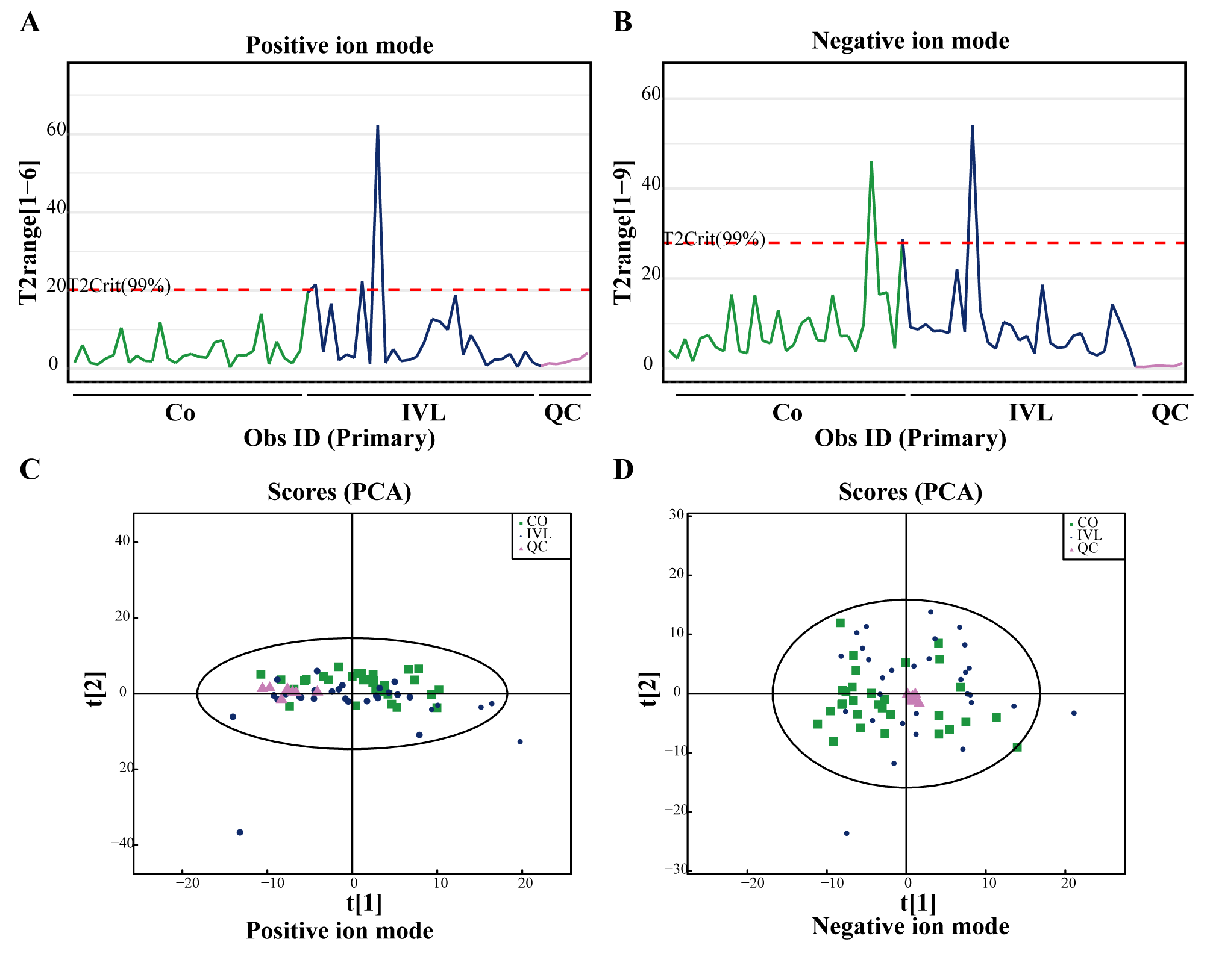

Supplement: Supplementary Figure 1 — Quality control analysis of the project. (A,B) Total ion chromatogram of all samples along with quality controls in positive and negative modes, respectively. The abscissa represented the retention time of each chromatographic peak, and the ordinate represented the peak intensity. (C,D) Principal component analysis of all samples PCA in the positive and negative modes, respectively. [file Image_1.TIF]
